# Genome Sequences of the Two *Telluria* Type Strains, Telluria chitinolytica ACM 3522^T^ and Telluria mixta DSM 29330^T^

**DOI:** 10.1128/mra.00215-23

**Published:** 2023-06-13

**Authors:** Andri Frediansyah, Henrike Miess, Ilse Cleenwerck, Harald Gross

**Affiliations:** a Research Group of Biotechnology and Microbial Metabolites for Food, National Research and Innovation Agency (BRIN), Yogyakarta, Indonesia; b PRTPP, Research Organization for Agriculture and Food, National Research and Innovation Agency (BRIN), Yogyakarta, Indonesia; c Department of Pharmaceutical Biology, Pharmaceutical Institute, University of Tübingen, Tübingen, Germany; d Laboratory of Microbiology and BCCM/LMG Bacteria Collection, Faculty of Sciences, Ghent University, Ghent, Belgium; Loyola University Chicago

## Abstract

High-quality draft genome sequences were obtained for the two type strains Telluria chitinolytica ACM 3522^T^ and Telluria mixta DSM 29330^T^. The genomes of both strains show a considerable biosynthetic potential to produce secondary metabolites.

## ANNOUNCEMENT

Gram-negative bacteria are known as a prolific source of natural products with unique chemical scaffolds and biological modes-of-action (1, 2). In the course of our ongoing screening for active secondary metabolites from Gram-negative bacteria from different genera (3–6), we are currently investigating the potential of the genus *Telluria*. The genus is formed by the two type strains, Telluria chitinolytica ACM 3522^T^ (= LMG 28806^T^) and Telluria mixta DSM 29330^T^ (= ACM 1762^T^ = LMG 11547^T^), which both represent soil bacteria (7, 8). *T. chitinolytica* was furthermore described to possess nematicidal properties (9). To reveal the genetic background of their beneficial properties and to shed light on the biosynthetic capacity for secondary metabolism, both type strains were sequenced. An Illumina-based whole-genome sequencing (WGS) project for Telluria mixta ACM 1762^T^ has been recently deposited at DDBJ/ENA/GenBank (accession number JANUHC000000000). However, it is quite fragmented, with 47 contigs, and therefore less suitable for genome mining for relatively large secondary metabolite gene clusters since it bears the risk that they will also be split up and not adequately recognized. This problem will be overcome with the PacBio long-read sequencing method.

In 2015, the *T. chitinolytica* strain was obtained from the Australian Collection of Microorganisms (ACM), while the *T. mixta* strain was obtained from the German Culture Collection (DSMZ). Cells were grown in liquid for 2 to 3 days at 27°C in 20 mL nutrient broth (NB) on a rotary shaker (180 rpm), inoculated directly from −80°C frozen stock, and then pelleted by centrifugation. For genomic DNA isolation, the Qiagen genomic DNA purification kit was used in combination with 100/G Genomic-tips according to the manufacturer’s protocol, except that for the bacterial lysis, the handled volumes were doubled, and the incubation time at 50°C was prolonged until a clear lysate was obtained. Macrogen, Inc. (Seoul, South Korea), generated the PacBio RS II data (Table 1) using the 8PAC V3, DNA polymerase binding kit P6, and one single-molecule real-time (SMRT) cell; a g-TUBE (Covaris, Inc., Woburn, MA, USA) was used for DNA shearing followed by BluePippin size selection (Sage Science, MA, USA). *De novo* assembly was performed utilizing HGAP3, whose protocol relies on PreAssembler v1 for filtering, PreAssembler v2 and AssembleUnitig v1 for assembly, BLASR v1 (10) for mapping, and Quiver v1 for consensus polishing. Default parameters were used except where otherwise noted. When the contig ends overlapped, contigs were connected to form a circular DNA. Annotation was performed with the NCBI Prokaryotic Genome Annotation Pipeline (PGAP, v6.4) pipeline (11, 12).

**TABLE 1:** Features of the complete whole-genome sequences of the two *Telluria* type strains

Statistic or characteristic	*T. chitinolytica* ACM 3522^T^	*T. mixta* DSM 29330^T^
PacBio sequencing		
Total no. of bases^*a*^	650,003,981	724,390,986
No. of reads^*b*^	72,494	85,268
Mean subread length (bp)	9,049	8,495
*N*_50_ (bp)^*c*^	12,921	12,236
Avg coverage (×)	103	97
*De novo* assembly		
Genome size (bp)	6,387,078	7,444,617
GC content (%)	66.3	65.9
No. of contigs	1 circular	1 circular
No. of gaps	0	0
Avg contig size (bp)	6,387,078	7,444,617
*N*_50_ (bp)^*d*^	6,387,078	7,444,617
No. of genes (total)	5,633	6,554
No. of genes (coding)	5,415	6,345
Specialized analyses		
No. of BGCs^*e*^	12	9

a Total number of bases in the subreads that passed filtering.

b Total number of reads that passed filtering.

c *N*_50_, 50% of all bases come from subreads longer than this value.

d *N*_50_, 50% of all filtered subreads are longer than the indicated value.

e BGCs, biosynthetic gene clusters coding for secondary metabolites, identified using antiSMASH v7.

The sequenced *Telluria* strains shared a similar G+C content of about 66% (Table 1), which is consistent with the genus description (67 to 72%) (7, 8). In comparison with the existing genome of *T. mixta* (7.39 Mbp), the genome size determined in our study appeared to be slightly larger (7.44 Mbp). Secondary metabolism analysis using antiSMASH 7.0 (13) predicted 9 to 12 biosynthetic gene clusters per strain (Table 1), revealing the encouraging potential of this genus for the production of novel bioactive compounds.

### Data availability.

GenBank accession numbers for raw sequencing data and genome assemblies are as follows: *T. chitinolytica* ACM 3522^T^, SRR23700812 and CP119083, and *T. mixta* DSM 29330^T^, SRR23715232 and CP119520, respectively.
